# Adverse events in meningioma surgery classified using the therapy-disability-neurology (TDN) grading system

**DOI:** 10.1007/s11060-025-05312-6

**Published:** 2025-12-16

**Authors:** Tim Leistner, Alexis Paul Romain Terrapon, Isabel Charlotte Hostettler, Oliver Bozinov, Anna Maria Zeitlberger, Marian Christoph Neidert

**Affiliations:** 1 Department of Neurosurgery, HOCH Health Ostschweiz, Kantonsspital St.Gallen, University Teaching and Research Hospital, St.Gallen, Switzerland; 2 Department of Neurosurgery, Inselspital, Bern University Hospital, Bern, Switzerland; 3 University of Zurich, Zurich, Switzerland

**Keywords:** TDN, Adverse event, AE, Meningioma, Surgery, Complications

## Abstract

**Purpose:**

Meningiomas are common, mostly benign, and often asymptomatic. Minimizing surgical adverse events (AE) is essential to maintain a favorable risk-benefit balance. Traditional AE grading systems often failed to account for disabling complications like neurologic deficits, which led to the development of the multidimensional therapy-disability-neurology grade (TDN). This study evaluates risk factors and consequences of AE in meningioma patients using TDN.

**Methods:**

Pre- and perioperative factors associated with the occurrence and severity of AE at discharge and follow-up were retrospectively identified in a monocentric cohort of consecutive patients undergoing surgery between 2013 and 2022. Significant variables of the univariable analysis were consequently tested in a multivariable analysis. Statistical analysis to detect the relationship between TDN and clinical outcomes was performed.

**Results:**

367 patients were included with a mean age at surgery of 60.8 years. A total of 95 AE at discharge and 144 AE at follow-up were recorded. Generalized linear models showed a relationship between the modified Rankin Scale on admission, tumor complexity as measured by the Milan Complexity Scale, and preoperative embolization with the frequency of AE at discharge and follow-up. A correlation between TDN, Karnofsky Performance Scale at discharge, and length of hospital stay was observed.

**Conclusion:**

The severity of AE as classified according to TDN correlated with the length of hospital stay and functional outcome following meningioma resection in our cohort and may be predicted by specific pre- and perioperative factors.

**Supplementary Information:**

The online version contains supplementary material available at 10.1007/s11060-025-05312-6.

## Introduction

Meningiomas are the most common primary intracranial tumors and are mostly classified as WHO CNS grade 1 [1]. Grade 2 and 3 meningiomas are rare and display a more rapid or aggressive growth. In addition to the WHO CNS grade, several molecular alterations and epigenetic patterns, including a CDKN2A/B homozygous deletion and loss of histone H3K27me3, have recently been associated with recurrence risk and prognosis [2–5]. Meningiomas are frequently incidental findings, and asymptomatic patients can often be followed without treatment. Symptomatic patients primarily present with headaches, neurologic deficits, or seizures and are primarily treated with surgery [6, 7]. Maximum-safe-resection may be followed by radiotherapy for high-grade tumors [7], while systemic therapy is not well established and remains an experimental option in cases that cannot be managed by surgery and radiotherapy.

Although the majority of patients benefit from surgery with symptomatic improvement or symptom resolution [6], meningioma resection carries a risk of adverse events (AE). The reported AE rates in the literature vary between 10 and 25% and include seizures, intracranial bleeding, thromboembolic events, infections, and new neurologic deficits [8, 9]. The classification of perioperative AE is essential to ensure the highest quality of clinical practice and research by monitoring and comparing quality of treatment.

The Clavien-Dindo-Grading system (CDG) has been the most commonly reported therapy-based classification proposed since 2004 [10, 11]. Originally designed for general surgery, it was adapted for neurosurgery in 2011 by Landriel *et a*l [12]. As both classifications are based on therapy required to counteract the AE, they fail to detect the severity of AE that cannot be treated. This is a very relevant limitation in meningioma surgery, which may result in focal neurologic deficits that may chronically impair the quality of life but are graded as minor AE in treatment-based classifications [13–16]. Therefore, a multidimensional grading system, the therapy-disability-neurology (TDN) grade, was introduced in 2021 to address these concerns in neurosurgery by adding scales of disability and neurologic deficits to previous classifications [17]. It was already suggested that introducing this system for the prediction of AE in meningioma surgery may lead to more comparable surgical results [18].

Here, we applied TDN to a retrospective, monocentric meningioma cohort and assessed its relationship with clinical, pathological, and radiological factors. The aim of this study was to identify preoperative factors that are associated with AE and assess the validity of TDN in this patient group.

## Methods

### Patients

We retrospectively evaluated medical records of all histologically verified meningiomas undergoing resection at the Cantonal Hospital St. Gallen between 2013 and 2022. Inclusion criteria for this study were: (1) histologically diagnosed meningioma; (2) age ≥ 18 years; (3) available Karnofsky Performance Scale (KPS) and modified Rankin scale (mRS) on admission and discharge; and (4) no rejection of the general consent.

### Measures

Data was retrospectively obtained from medical records. Length of hospital stay (LOS) was measured from surgery to discharge. AE were collected for discharge, follow-up 3 months after surgery, and were classified according to TDN. Major adverse events (mAE) were defined as a new TDN score ≥ 3, and minor AE as TDN score 1 or 2. Karnofsky Performance Scale, mRS, and National Institute of Health Stroke Scale (NIHSS) were collected on admission, at discharge, and at follow-up 3 months after surgery. Discharge modalities (home, rehabilitation facility, external hospital) were obtained for clinical records. For univariable and multivariable logistic analysis of prediction factors for TDN, the following parameters were extracted: American Society of Anesthesiologists risk classification (ASA), age, biological sex, number of tumors, different therapies, number of surgeries, location, surgery type, surgical priority, surgery duration, blood loss, Simpson grade, and Milan Complexity Score [19]. Histological information was collected from neuropathological reports of board-certified neuropathologists, including WHO grade, MIB-1/Ki-67 index, microscopically visible brain invasion, necrosis, increased cellularity, nucleus-to-cytoplasmic ratio, nucleoli, and patternless growth. MRI and CT reports from board-certified radiologists were scanned for information on maximum diameter and the following radiological features: edema, bone infiltration, cystic tumor parts, hyperostosis, and calcifications. Smoking status, cortisone treatment, and clinical symptoms such as anamnestic mental alterations were retrospectively collected from medical records. Tumor volumes were measured using the Brainlab Elements software and its SmartBrush application (Brainlab AG, Munich).

### Statistical analysis

The programming language R (version 2025.09.1 + 401) was used to perform statistical analysis. Medians are presented with interquartile range (IQR), means with standard deviations (SD), and percentages with 95% confidence intervals (CI, Sison-Glaz when multinomial and Wilson method when binomial). We analyzed the differences between groups with and without AE with Student’s t-test for normally distributed variables, reported Cohen’s *d* as effect size, or the Wilcoxon rank sum test for distribution-free variables. Chi-Square Tests were used to assess nominal variables and are reported with odds ratios (OR), 95% confidence intervals, and X^2^ (Supplementary Table 1) [20]. Correlations were reported with Spearman’s rank correlation coefficient (rho, with proportion of shared variance R^2^) or Kendall`s Tau for variables with many tied ranks. Weak correlations were defined as Tau 0.06–0.26 or rho 0.10–0.39, and moderate correlations as Tau 0.27–0.49 or rho 0.40–0.70. Numbers for missing data were reported, and patients were excluded from the primary analysis. A sensitivity analysis was performed after multivariate imputation by chain equations for missing data (Supplementary Tables 2 & 3) [21]. Patients with AE at discharge and lost to follow-up were included in AE at follow-up, and TDN passed on. Differences between TDN and interval/ordinal variables were explored with the Kruskal-Wallis rank sum test, and Dunn’s post hoc multiple comparison was used to compare the mean rank of each group with each other. Linear multiple regression models were reported with relative importance of each predictor normalized to the sum of 100%. Preoperative variables associated with AE/TDN were identified, and significant variables were tested in a multivariable analysis. Generalized linear models were reported with McFadden shared variance R^2^ and effect size of TDN compared to CDG reported with Cohen’s *f*^2^ [20]. Level of statistical significance was set to < 0.05. We report both the original p-values and the Benjamini-Hochberg procedure (BH) adjusted p-values for multiple comparison correction (Supplementary Tables 4 & 5) [22]. The findings of this paper were reported in accordance with the Strengthening the Reporting of Observational Studies in Epidemiology (STROBE) statement [23].

## Results

**Fig. 1:**
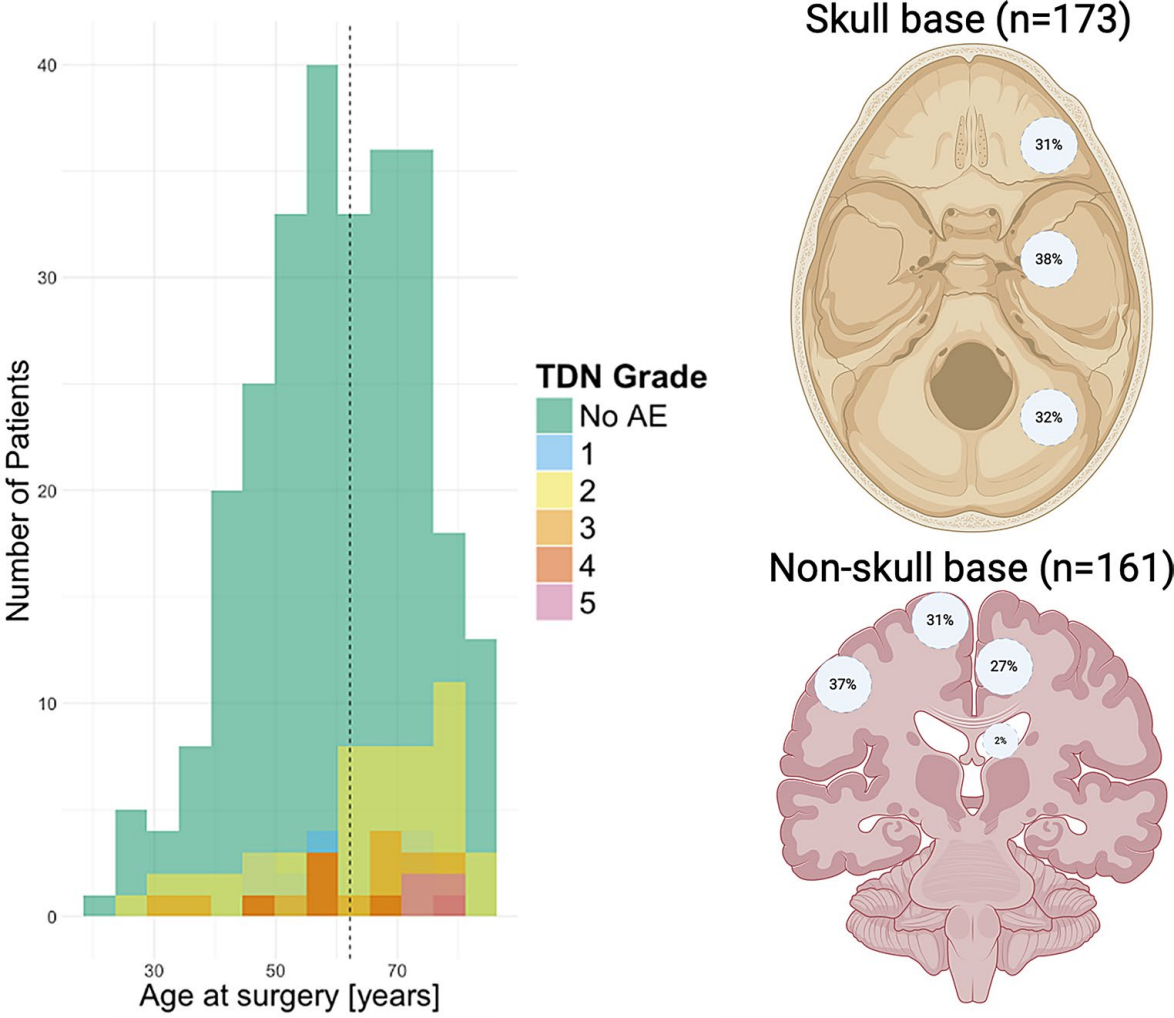
Age distribution and tumor location

We included all 367 patients in our registry undergoing treatment with a mean age of 60.8 years (SD 13.6) (Table 1; Fig. 1). We recorded 95 AE at discharge (25.9% [21.7–30.6]) and 144 AE at follow-up (*n* = 361, 39.9% [35.0–45.0]). There was a significant difference in mean age between patients without AE (59.8 [13.5]) and with AE (63.6 [13.7]) at discharge, *p* = 0.0200, *d* = 0.28. However, at follow-up, the mean age did not differ significantly between patients without AE (60.0 [13.5]) and patients with AE (61.6 [13.9]), *p* = 0.2669, *d* = 0.12. KPS on admission was significantly lower for patients with an AE (median 70 [20.0]) compared to those without an AE (80 [20.0]), *p* = 0.0010.

**Table 1:** Patient characteristics

**Sex**		
Female	256 (71.7%)	
Male	111 (28.3%)	
**Age**	60.8 (23.0-85.8)	
**Tumor location**		
Left	170 (46.3%)	
Right	150 (40.9%)	
Both sides	47 (12.8%)	
Cranial	334 (91.0%)	
Spinal	33 (9.0%)	
**Cranial location**		
Skull base	173 (47.1%)	
Convexity	109 (29.7%)	
Falx	44 (12.0%)	
Intraventricular	4 (1.1%)	
Intraosseous	4 (1.1%)	
**WHO CNS grading**		
Grade 1	324 (88.3%)	
Grade 2	40 (10.9%)	
Grade 3	3 (0.8%)	
**Adjuvant and neoadjuvant therapy**		
No therapy	320 (87.2%)	
Radio therapy	39 (10.6%)	
Embolization	11 (3.0%)	
Systemic therapy	3 (0.8%)	
**Cardinal symptom**		
Focal neurologic deficit	141 (38.4%)	
Asymptomatic	60 (16.3%)	
Seizure	49 (13.4%)	
Headache	47 (12.8%)	
Mental alteration	27 (7.4%)	
Other	43 (11.7%)	
**Clinical scores**	**Admission**	**Follow-up**
mRS	1.0 (0–5)	1.0 (0–6)
KPS Score	80.0 (20–100)	90.0 (0-100)
NIHSS	0.0 (0–21)	0.0 (0–38)
**Tumor volume**		
Total tumor volume	13.1 (0-222.8)	
Max. diameter	3.2 (0–13.0)	
**TDN**	**Discharge**	**Follow-up**
1	13 (3.5%)	19 (5.2%)
2	54 (14.7%)	60 (17.0%)
3	18 (4.9%)	51 (14.5%)
4	6 (1.6%)	8 (2.3%)
5	4 (1.1%)	6 (1.7%)

Clinical and tumor-specific characteristics, number of patients with individual characteristics are displayed with percentages; age, clinical scores, diameter, and volume are presented with median (Minimum-Maximum). NIHSS = National Institute of Health Stroke Scale, KPS = Karnofsky Performance Scale, mRS = modified Rankin Scale, TDN = therapy-disability-neurology grading system

Age distribution of included meningioma patients and therapy-disability-neurology grading (TDN) at discharge, the dotted black line indicates median age. Distribution of skull base meningioma in anterior, medial, and posterior fossa. Non-skull base meningiomas are sorted in decreasing order: convexity meningiomas, paramedian and parasagittal (with contact to superior sagittal sinus), falcine, and intraventricular. Intraosseous meningiomas are not depicted. Created in BioRender. Neidert, M. (2025) https://BioRender.com/h4nm559. AE = adverse events.

### Preoperative factors association with AE and mAE

Preoperative factors significantly associated with AE at discharge were preoperative embolization, mRS ≥ 2 on admission, MCS ≥ 4, age ≥ 60 years, and recurrent and multiple tumors. AE at follow-up were additionally significantly associated with preoperative seizures, cranial tumors, radiation-induced tumors, and male sex; however, age ≥ 60 years was no longer significantly associated (Table 2). Mean diameter of embolized tumors (6.2 cm [2.9]) was significantly larger than non-embolized tumors (3.5 cm [1.9]), *p* = 0.001, *d* = 1.41. Major AE were associated with preoperative mental alterations and recurrent tumors (Table 2). We found no significant association between different intracranial locations, such as skull base, relationship to the tentorium, the superior sagittal sinus, radiological features, or smoking status (Supplementary Tables 6 & 7).

**Table 2:** Pre- and perioperative risk factors associated with AE (TDN)

**Pre- and perioperative factors**	**TDN at discharge**	**TDN at follow-up**	
ASA	T = 0.09, *p* = 0.0673	T = 0.10, *p* = 0.0335*	
MCS	T = 0.15, *p* = 0.0012*	T = 0.13, *p* = 0.0052*	
NIHSS	T = 0.11, *p* = 0.0261*	T = 0.10, *p* = 0.0400*	
KPS	T = -0.15, *p* = 0.0006*	T = -0.16, *p* = 0.0004*	
Age	*R* = 0.14, *p* = 0.0065*	*R* = 0.07, *p* = 0.1903	
Simpson grade	T = 0.21, *p* < 0.0001*	T = 0.11, *p* = 0.0159*	
Tumor diameter	*R* = 0.20, *p* = 0.0001*	*R* = 0.17, *p* = 0.0017*	
Tumor volume	*R* = 0.19, *p* = 0.0045*	*R* = 0.14, *p* = 0.0155*	
Blood loss	*R* = 0.17, *p* = 0.0024*	*R* = 0.16, *p* = 0.0042*	
Surgery duration	*R* = 0.22, *p* < 0.0001*	*R* = 0.26, *p* < 0.0001*	
	**Adverse events at discharge**	**Adverse events at follow-up**	**Major adverse events**
Multiple tumors	OR = 2.20 (1.13–4.23, *p* = 0.0100)	OR = 2.33 (1.23–4.45, *p* = 0.0046)*	OR = 3.94 (1.45–13.43, *p* = 0.0033)
Embolization	OR = 3.58 (0.89–15.24, *p* = 0.0276)	OR = 4.18 (0.98–24.88, *p* = 0.0239)	OR = 1.22 (0.03–9.16, *p* = 0.8529)
Mental alterations	OR = 1.64 (0.86–3.07, *p* = 0.0954)	OR = 1.58 (0.87–2.85, *p* = 0.1040)	OR = 3.72 (1.48–9.01, *p* = 0.0008)*
Seizure	OR = 1.38 (0.72–2.57, *p* = 0.2809)	OR = 1.85 (1.03–3.33, *p* = 0.0258)	OR = 1.03 (0.29–2.93, *p* = 0.9516)
Age ≥ 60	OR = 1.69 (1.02–2.82, *p* = 0.0314)	OR = 1.29 (0.82–2.01, *p* = 0.2436)	OR = 1.61 (0.68–4.02, *p* = 0.2415)
MCS ≥ 4	OR = 1.90 (1.10–3.33, *p* = 0.0143)	OR = 1.83 (1.13–3.00, *p* = 0.0093)*	OR = 2.08 (0.81–6.00, *p* = 0.1006)
mRS ≥ 2	OR = 2.02 (1.21–3.36, *p* = 0.0039)*	OR = 1.91 (1.19–3.06, *p* = 0.0042)*	OR = 2.13 (0.91–5.02, *p* = 0.0502)
Male sex	OR = 1.60 (0.95–2.69, *p* = 0.0594)	OR = 1.58 (0.98–2.56, *p* = 0.0461)	OR = 1.10 (0.42–2.66, *p* = 0.8201)
Recurrent tumors	OR = 2.41 (1.21–4.76, *p* = 0.0052)*	OR = 2.51 (1.29–4.98, *p* = 0.0031)*	OR = 3.06 (1.09–7.88, *p* = 0.0094)
Radiation induced tumors	OR = 1.66 (0.35–6.71, *p* = 0.4205)	OR = 3.63 (0.81–22.13, *p* = 0.0486)	OR = 2.81 (0.28–14.62, *p* = 0.1807)
WHO CNS grade ≥ 2	OR = 1.50 (0.70–3.13, *p* = 0.2416)	OR = 1.59 (0.79–3.20, *p* = 0.1546)	OR = 2.88 (0.97–7.70, *p* = 0.0191)
Brain invasion	OR = 2.30 (0.33–13.97, *p* = 0.2691)	OR = 2.19 (0.36–15.28, *p* = 0.2970)	OR = 6.67 (0.59–45.16, *p* = 0.0125)
Postoperative cortisone	OR = 2.47 (1.46–4.25, *p* = 0.0003)*	OR = 2.56 (1.61–4.11, *p* < 0.0001)*	OR = 2.38 (0.90–7.03, *p* = 0.0558)
Simpson grade ≥ 3	OR = 3.71 (2.22–6.26, *p* < 0.0001)*	OR = 2.09 (1.31–3.34, *p* = 0.0010)*	OR = 2.05 (0.87–4.82, *p* = 0.0640)
Cranial tumors	OR = 2.71 (0.91–10.90, *p* = 0.0584)	OR = 3.94 (1.45–13.43, *p* = 0.0033)*	OR = 2.81 (0.43–118.73, *p* = 0.2968)

Pre- and perioperative factors significantly associated with adverse events (AE) and therapy-disability-neurology grading (TDN) at discharge or follow-up, and major adverse events (mAE). Correlations are displayed with Kendall’s Tau (T), Spearman’s rank correlation coefficient (R), and associations are reported as odds ratios (OR) with confidence intervals. ASA = American Society of Anesthesiologists risk classification, MCS = Milan Complexity Score, NIHSS = National Institute of Health Stroke Scale, KPS = Karnofsky Performance Scale, mRS = modified Rankin Scale, ^(^*^)^ indicates significance after the Benjamini-Hochberg procedure

### Correlation with TDN

**Fig. 2:**
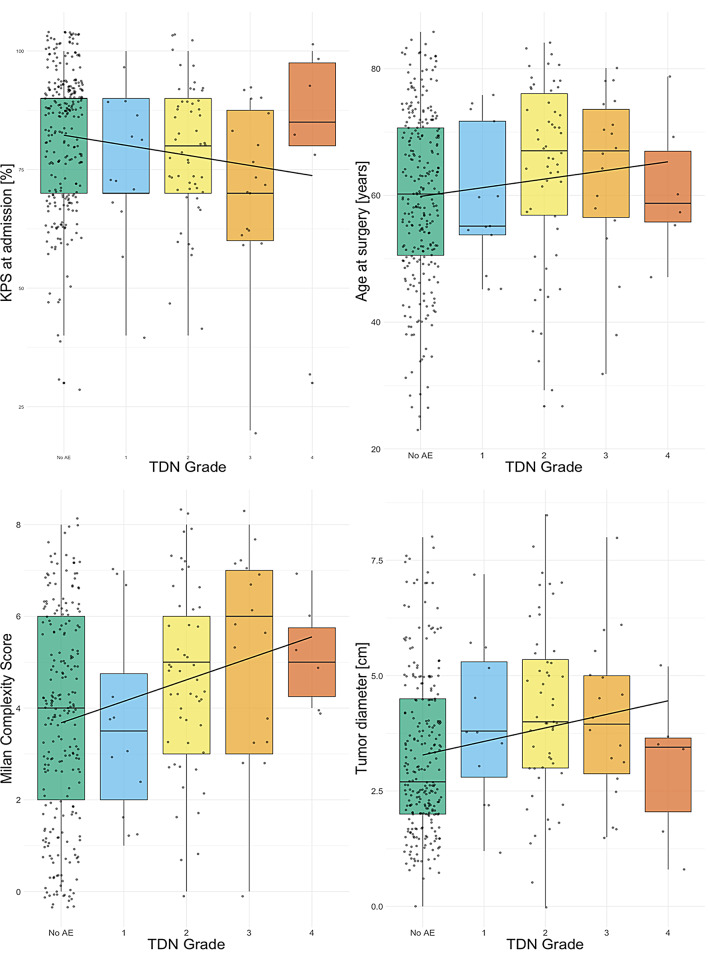
Relationship between therapy-disability-neurology grading (TDN) and preoperative variables

TDN at discharge significantly correlated with MCS, age, KPS on admission, NIHSS on admission, tumor diameter, and volume (Fig. 2, Supplementary Fig. 1). For follow-up TDN, we additionally found a significant correlation for ASA; however, age was no longer significant (Table 2). ASA was significantly correlated with TDN at discharge and follow-up in cranial meningioma (Supplementary Table 4).

### Perioperative factors association with AE and mAE

Perioperative factors such as Simpson grade, blood loss, histological brain invasion, WHO CNS grade ≥ 2, surgery duration, and postoperative treatment with corticosteroids were associated with AE/TDN at discharge, follow-up, or mAE (Table 2). We found no significant association between AE and histological features, except for brain invasion with mAE (Supplementary Tables 6 & 7).

TDN 5 (death) was excluded. The central line within each box represents the median for each TDN, while the boxes show the interquartile range (IQR), whiskers extend to 1.5 times the IQR. A linear regression line (solid black) is included. Individual data is visualized as dots and jittered for better interpretability. Max. diameter was capped at 8.6 cm, excluding 6 measurements. KPS = Karnofsky Performance Scale.

### Multivariable analysis

Multivariable analysis was performed for preoperative variables that were significantly correlated with TDN/AE at discharge, follow-up, and for mAE in cranial tumors (Supplementary Table 4). Spinal tumors were excluded as MCS is specific for cranial tumors. Diameter was selected for multivariable analysis and volume was excluded due to missing values (mainly because of lacking high resolution MRI scans). Significant variables for the prediction of AE at discharge in generalized linear models were MCS ≥ 4, mRS ≥ 2, and preoperative embolization (Table 3, discharge: McFadden R^2^ = 8.7%, follow-up: McFadden R^2^ = 7.2%). A generalized linear model for the prediction of mAE showed significant prediction of mental alterations and recurrent tumors (McFadden R^2^ = 15.2%).

**Table 3:** Generalized linear models for the prediction of AE

Generalized linear model factors	Adverse events at discharge
Multiple tumors	OR = 1.26 (0.76–2.07, *p* = 0.3604)
Embolization	OR = 2.69 (1.09–6.91, *p* = 0.0309)
Age ≥ 60	OR = 1.41 (0.96–2.07, *p* = 0.0801)
MCS ≥ 4	OR = 1.54 (1.06–2.28, *p* = 0.0274)
mRS ≥ 2	OR = 1.62 (1.11–2.37, *p* = 0.0126)
Recurrent tumors	OR = 1.60 (0.95–2.66, *p* = 0.0707)
Headache	OR = 0.73 (0.44–1.16, *p* = 0.1897)
	**Adverse events at follow-up**
Multiple tumors	OR = 1.41 (0.87–2.29, *p* = 0.1611)
Embolization	OR = 2.86 (1.14–8.64, *p* = 0.0349)
ASA ≥ 3	OR = 1.28 (0.89–1.82, *p* = 0.1783)
MCS ≥ 4	OR = 1.54 (1.10–2.17, *p* = 0.0131)
mRS ≥ 2	OR = 1.55 (1.08–2.23, *p* = 0.0163)
Recurrent tumors	OR = 1.50 (0.92–2.46, *p* = 0.1052)
	**Major adverse events**
Mental alterations	OR = 3.48 (1.52–8.26, *p* = 0.0033)
Recurrent tumors	OR = 2.67 (0.97–6.98, *p* = 0.0468)
Brain invasion	OR = 2.23 (0.38–13.34, *p* = 0.3578)
mRS ≥ 2	OR = 1.51 (0.66–3.45, *p* = 0.3185)
WHO CNS grade ≥ 2	OR = 1.09 (0.25–3.64, *p* = 0.8915)

Generalized linear models predicting adverse events at discharge (*n* = 326), follow-up (*n* = 322), and major adverse events (*n* = 255). Individual risk factors included in the analysis are displayed, and the odds ratios with confidence intervals are presented. ASA = American Society of Anesthesiologists risk classification, MCS = Milan complexity Score, mRS = modified Rankin Scale

### Relationship between TDN and outcomes

At discharge, 199 patients (54.2% [49.0–59.6]) were discharged home, 161 (43.9% [38.7–49.3]) to a rehabilitation facility, 3 (0.8% [0.0–6.2]) to different, usually the referring hospitals, and 4 (1.1% [0.0–6.5]) patients died. Median length of hospital stay was 8 (4.0). Patients with documented AE at discharge had significantly longer hospitalizations (median = 10 [6.0]) than those without AE (median = 7 [2.3], *p* < 0.0001) and led to more frequent discharges to rehabilitation facilities (no AE: 35.3% [29.8–41.4], AE: 68.4% [60.0–78.3], *p* < 0.0001). LOS significantly increased with increasing TDN compared to TDN 0, except for grade 5 (death) and grade 1 (*p* < 0.0001, Supplementary Table 8), and led to an increased frequency of discharge to rehabilitation facilities (*p* < 0.0001, Supplementary Table 9). Moderate correlation between LOS and TDN was observed (rho = 0.42, R^2^ = 17.77%, *p* < 0.0001, Supplementary Figs. 2 & 3). TDN’s most important dimension for predicting LOS was therapy. The multiple regression model excluding TDN 5 showed a relative importance of 64% for therapy, 26% for disability, and 10% for neurology (multiple R^2^ = 49.61%, *p* < 0.0001).

Median KPS at discharge was 90 (20). KPS at discharge and follow-up was significantly lower for patients who had a documented AE at discharge (median: 70 (30), follow-up: median: 90 (20), *p* < 0.0001). KPS at discharge and at follow-up was negatively affected by TDN compared to TDN 0, except for grade 1, *p* < 0.0001. KPS at discharge and follow-up showed moderate correlation (discharge: tau = -0.33, follow-up: tau = -0.27, *p* < 0.0001). The multiple regression model for TDN as a predictor and KPS at discharge as the outcome variable, excluding TDN 5, showed relative importance: therapy = 45%, disability = 40% and neurology = 15% (multiple R^2^ = 24.06%, *p* < 0.0001). The linear regression model of TDN, excluding TDN 5, as a predictor for LOS and KPS at discharge was also significant, with a lower R^2^ than the multiple regression models (LOS: multiple R^2^ = 43.25%, KPS: multiple R^2^ = 20.46%, *p* < 0.0001).

Linear regression models of CDG, excluding grade 5 (death), as a predictor for LOS and KPS at discharge were significant with lower proportions of variance explained compared to TDN (LOS: multiple R^2^ = 30.93%, KPS: multiple R^2^ = 17.18%, *p* < 0.0001). TDN compared to CDG for the prediction of LOS showed a medium increase in effect size, Cohen’s *f*^2^ = 0.22 (TDN: multiple R^2^ = 43.25%, CDG: multiple R^2^ = 30.93%), and a small increase in effect size for the prediction of KPS, Cohen’s *f*^2^ = 0.04 (TDN: multiple R^2^ = 20.46%, CDG: multiple R^2^ = 17.18%).

### Sensitivity analysis

Sensitivity analysis was performed for missing values and showed consistent significance and direction for all variables except for smoking status at discharge and follow-up (discharge: OR = 1.25 [0.70–2.21], *p* = 0.4139, discharge imputed: OR = 1.89 [1.13–3.15], *p* = 0.0157, follow-up: OR = 1.47 [0.87–2.47], *p* = 0.1249, follow-up imputed: OR = 1.72 [1.11–2.67], *p* = 0.0198, Supplementary Table 3).

## Discussion

TDN was a consistent measure for the severity of AE in meningioma surgery at our institution. Adverse events classified by TDN were significantly associated with increased LOS, discharge to rehabilitation facilities, and, accordingly, functional impairment at discharge and follow-up. Risk factors such as ASA score, MCS, NIHSS, KPS at admission, tumor diameter and volume, Simpson grade, blood loss, and surgery duration significantly correlated with TDN at follow-up; however, correlations were weak to moderate in strength. Explanatory power of TDN for outcome measures may be superior to CDG for LOS and for KPS.

Prospective patient registries are implemented in many neurosurgical centers for quality control and research on outcomes [13, 19, 24–28]. However, the limitations of existing AE grading systems and the need for a standardized and neurosurgery-specific classification of AE is evident [18, 24, 29–34].

In meningioma surgery and neurosurgery in general, the standardized reporting of AE is of paramount importance to monitor, compare, and improve the quality of treatment, as well as to address research questions. Unfortunately, standardized reporting of AE in meningioma surgery is lacking. The introduction of the Clavien-Dindo classification for general surgery greatly harmonized the reporting of AE [10, 11, 35, 36]. Various other classification systems have been proposed for neurosurgical purposes aiming to achieve a similar consistency [12, 30, 37–39], but none have gained wide acceptance. The two most frequently used neurosurgery-specific grading systems are the Landriel Ibanez Classification (LIC) and the Spinal Adverse Events Severity System (SAVES-V2) [12, 39].

Both however do not grade the severity of the disability, which is probably the most important aspect for patients suffering from AE.

Unlike any previous classification systems, TDN does not only take into account the used therapies, but also the disability grade and neurologic deficits. Corresponding to the CDG classification the “Therapy” TDN T1 to T5 equals CDG grade 1 to 5 [10]. The “Disability” dimension grades the severity of the functional impairment caused by the AE and is also graded from 1 to 5 based on the mRS [40]. Although the “Disability” dimension already incorporates disabling neurologic deficits, the “Neurology” dimension was added as some neurologic deficits may cause distress without being disabling, such as a severe facial paresis (N1 = no new neurologic deficit, N2 = new neurologic deficit). Importantly, TDN not only summarizes the severity of AE from 1 to 5, making it easy to be used for complication analyses, but also provides a separate grading for each dimension, offering a nuanced understanding of each AE.

Since its introduction in 2021, TDN has already been implemented in several institutions and patient cohorts [26, 32, 41–43]. Gómez Vecchio et al. compared TDN to the LIC in a cohort of 231 patients with diffuse lower-grade gliomas, of which 110 suffered AE [32]. They outlined that TDN captured more AE of higher severity compared to the LIC. They also demonstrated that a new neurologic deficit correlates with a deterioration of quality of life; however, this was not captured by TDN rather by specific deficits in daily activities. Li et al. used TDN to classify complications in patients >65 years undergoing meningioma resection and showed that AE of higher severity were associated with increased mortality [41]. In summary, it has been previously shown that TDN correlates with functional outcome (KPS) [17], mortality [41], costs [41], as well as LOS [17]. An association with quality of life still has to be explored in a larger cohort.

TDN was recently validated using a survey-based approach, demonstrating substantial to almost-perfect inter- and intra-observer reliability. It was well received by the neurosurgical community, with the vast majority recommending its adoption in future literature [44].

This study is limited by its retrospective and monocentric design. The retrospective design may introduce temporal bias over a time span of nine years, during which surgical techniques, perioperative care standards, and imaging technology for volumetry have evolved. Future surgical registries should include quality-of-life outcomes to better assess the impact of AE and to better assess the clinical relevance and validity of TDN. We aim at an external validation of our findings with a larger multicentric cohort of meningioma patients and a systematic review of risk factors associated with postoperative AE.

## Conclusion

There is no widely accepted classification of AE in meningioma and in neurosurgery in general. We therefore introduce TDN as a grading system measuring AE severity in meningioma surgery. Severity and occurrence of AE when classified according to TDN may be predicted by specific pre- and perioperative factors. TDN correlates with length of hospital stay and functional outcome following neurosurgery and was therefore shown to be a useful tool in meningioma surgery in our cohort.

## Supplementary Information

Below is the link to the electronic supplementary material.

Supplementary Material 1

## Data Availability

All raw data is available from the corresponding author upon reasonable request.
