# Supplementary material for: Adverse events in meningioma surgery classified using the therapy-disability-neurology (TDN) grading system
Source: J Neurooncol. 2025 Dec 16;176(2):125. doi: 10.1007/s11060-025-05312-6 (PMC12708695; doi:10.1007/s11060-025-05312-6)
Supplement: Supplementary file 1 — Supplementary Material 1 [file 11060_2025_5312_MOESM1_ESM.pdf]

Adverse events in meningioma surgery classified using the Therapy-Disability-Neurology (TDN) grading system

Journal of Neuro-Oncology

Tim Leistner, Alexis Paul Romain Terrapon, Isabel Charlotte Hostettler, Oliver Bozinov, Anna Maria Zeitlberger, Marian Christoph Neidert

Corresponding Author:

Marian Christoph Neidert

Department of Neurosurgery, HOCH Health Ostschweiz, Kantonsspital St.Gallen, University teaching and research hospital

Rorschacher Strasse 95, 9007 St.Gallen, Switzerland

Marian.neidert@h-och.ch

Supplementary Material 1

Table 1 Pre- and perioperative risk factors associated with AE reported with X^2

	Adverse events at discharge	Adverse events at follow-up	Major adverse events
Multiple tumors	$X^2 = 6.64, p = 0.0010$***	$X^2 = 8.03, p = 0.0046$**	$X^2 = 0.00, p = 0.9863$
Embolization	$X^2 = 4.86, p = 0.0276$*	$X^2 = 5.10, p = 0.0239$*	$X^2 = 0.03, p = 0.8529$
Mental alterations	$X^2 = 2.78, p = 0.0954$	$X^2 = 2.64, p = 0.1040$	$X^2 = 11.27, p = 0.0008$***
Seizure	$X^2 = 1.16, p = 0.2809$	$X^2 = 4.97, p = 0.0258$*	$X^2 = 0.00, p = 0.9516$
Age ≥ 60	$X^2 = 4.63, p = 0.0314$*	$X^2 = 1.36, p = 0.2436$	$X^2 = 1.37, p = 0.2415$
MCS ≥ 4	$X^2 = 6.00, p = 0.0143$*	$X^2 = 6.77, p = 0.0093$**	$X^2 = 2.70, p = 0.1006$
mRS ≥ 2	$X^2 = 8.35, p = 0.0039$**	$X^2 = 8.21, p = 0.0042$**	$X^2 = 3.84, p = 0.0502$
sex	$X^2 = 3.56, p = 0.0594$	$X^2 = 3.98, p = 0.0461$*	$X^2 = 0.05, p = 0.8201$
Recurrent tumors	$X^2 = 7.80, p = 0.0052$**	$X^2 = 8.73, p = 0.0031$**	$X^2 = 6.75, p = 0.0094$**
Radiation induced tumors	$X^2 = 0.65, p = 0.4205$	$X^2 = 3.89, p = 0.0486$*	$X^2 = 1.79, p = 0.1807$
WHO CNS grade ≥ 2	$X^2 = 1.13, p = 0.2877$	$X^2 = 2.59, p = 0.1077$	$X^2 = 5.50, p = 0.0191$*
Brain invasion	$X^2 = 1.22, p = 0.2691$	$X^2 = 1.09, p = 0.2970$	$X^2 = 6.24, p = 0.0125$*
Postoperative cortisone	$X^2 = 12.98, p = 0.0003$***	$X^2 = 17.64, p < 0.0001$***	$X^2 = 3.66, p = 0.0558$
Simpson grade ≥ 3	$X^2 = 29.62, p < 0.0001$***	$X^2 = 10.83, p = 0.0010$***	$X^2 = 3.43, p = 0.0641$
Cranial tumors	$X^2 = 3.58, p = 0.0585$	$X^2 = 8.62, p = 0.0033$**	$X^2 = 1.09, p = 0.2968$
ASA ≥ 3	$X^2 = 2.05, p = 0.1527$	$X^2 = 3.14, p = 0.0766$	$X^2 = 2.18, p = 0.1401$
Necrosis	$X^2 = 0.05, p = 0.8217$	$X^2 = 0.02, p = 0.8945$	$X^2 = 0.02, p = 0.8846$
Increased cellularity	$X^2 = 0.76, p = 0.3837$	$X^2 = 2.18, p = 0.1400$	$X^2 = 0.17, p = 0.6801$
High n/c ratio	$X^2 = 0.16, p = 0.6937$	$X^2 = 0.02, p = 0.8907$	$X^2 = 0.03, p = 0.8724$
Nucleoli	$X^2 = 0.00, p = 0.9931$	$X^2 = 0.96, p = 0.3282$	$X^2 = 0.00, p = 0.9845$
Patternless growth	$X^2 = 0.20, p = 0.6578$	$X^2 = 0.16, p = 0.6912$	$X^2 = 0.24, p = 0.6237$
Symptomatic tumors	$X^2 = 3.18, p = 0.0747$	$X^2 = 2.26, p = 0.1328$	$X^2 = 0.70, p = 0.4015$
Headache	$X^2 = 3.10, p = 0.0781$	$X^2 = 0.98, p = 0.3224$	$X^2 = 0.53, p = 0.4686$
Focal neurological deficits	$X^2 = 1.48, p = 0.2241$	$X^2 = 0.12, p = 0.7329$	$X^2 = 3.38, p = 0.0661$
Preoperative cortisone	$X^2 = 0.18, p = 0.6692$	$X^2 = 0.44, p = 0.5066$	$X^2 = 2.42, p = 0.8320$
Calcifications	$X^2 = 0.23, p = 0.6336$	$X^2 = 1.96, p = 0.1612$	$X^2 = 1.29, p = 0.2562$
Edema	$X^2 = 0.76, p = 0.3848$	$X^2 = 1.98, p = 0.1597$	$X^2 = 0.09, p = 0.7605$
Bone infiltration	$X^2 = 2.12, p = 0.1454$	$X^2 = 3.39, p = 0.065$	$X^2 = 2.01, p = 0.1561$
Cysitic tumors	$X^2 = 0.01, p = 0.9248$	$X^2 = 0.63, p = 0.4263$	$X^2 = 0.51, p = 0.4733$
Parenchymal infiltration	$X^2 = 2.60, p = 0.1069$	$X^2 = 0.88, p = 0.3472$	$X^2 = 0.26, p = 0.6128$
Hyperostosis	$X^2 = 0.03, p = 0.8622$	$X^2 = 1.05, p = 0.3056$	$X^2 = 1.16, p = 0.2825$
Nicotine	X2 = 0.67, $p = 0.4139$	X2 = 2.35, $p = 0.1249$	X2 = 0.16, $p = 0.6891$

Pre- and perioperative factors association with adverse events and therapy-disability-neurology grading (TDN) at discharge or follow-up (n = 361), and major adverse events reported with Chi-Square (X^2). ASA = American Society of Anesthesiologists risk classification, MCS = Milan Complexity Score, NIHSS = National Institute of Health Stroke Scale, KPS = Karnofsky Performance Scale, mRS = modified Rankin Scale, n/c ratio = nucleus-to-cytoplasmic ratio. Significance codes: '***' $p \leq 0.001$, '**' $p \leq 0.01$, '*' $p < 0.05$.

Table 2 Missing values for the full cohort and cranial tumors

Pre- and perioperative factors full cohort (n = 367)	Discharge	Follow-Up
MCS	n = 326	n = 322
NIHSS	n = 366	n = 360
Tumor diameter	n = 357	n = 352
Tumor volume	n = 282	n = 280
Blood loss	n = 308	n = 303
MCS ≥ 4	n = 326	n = 322
Postoperative cortisone	n = 352	n = 346
Surgery duration	n = 298	n = 293
Ki-67	n = 321	n = 311

Necrosis	n = 287	n = 283
Brain invasion	n = 276	n = 272
Increased cellularity	n = 278	n = 274
High n/c ratio	n = 278	n = 274
Nucleoli	n = 277	n = 273
Patternless growth	n = 281	n = 277
Preoperative cortisone	n = 349	n = 343
Calcifications	n = 343	n = 338
Edema	n = 348	n = 343
Bone infiltration	n = 349	n = 344
Cysitic tumors	n = 346	n = 341
Parenchymal infiltration	n = 346	n = 341
Hyperostosis	n = 346	n = 341
Nicotine	n = 335	n = 331
**Cranial pre- and perioperative factors (n = 334)**		
MCS	n = 326	n = 322
NIHSS	n = 333	n = 328
Tumor diameter	n = 327	n = 323
Tumor volume	n = 272	n = 270
Blood loss	n = 280	n = 276
Surgery duration	n = 269	n = 265
MCS ≥ 4	n = 326	n = 317
Postoperative cortisone	n = 320	n = 315
Nicotine	n = 306	n = 302

Factors with missing data at discharge and follow-up for the full cohort and cranial tumors. ASA = American Society of Anesthesiologists risk classification, MCS = Milan complexity Score, NIHSS = National Institute of Health Stroke Scale, n/c ratio = nucleus-to-cytoplasmic ratio.

Table 3 Multivariate Imputation by Chained Equations for pre- and perioperative risk factors

**Pre- and perioperative factors**	**TDN at discharge**	**TDN at discharge imputed**	**TDN at follow-up**	**TDN at follow-up imputed**		
MCS	T = 0.15, *p* = 0.0012	T = 0.14, *p* = 0.0015	T = 0.13, *p* = 0.0052	T = 0.12, *p* = 0.0058		
Tumor diameter	R = 0.20, *p* < 0.0001	R = 0.19, *p* = 0.0002	R = 0.17, *p* = 0.0017	R = 0.16, *p* = 0.0019		
Tumor volume	R = 0.19, *p* = 0.0045	R = 0.17, *p* = 0.0014	R = 0.14, *p* = 0.0155	R = 0.17, *p* = 0.0012		
Blood loss	R = 0.17, *p* = 0.0024	R = 0.20, *p* = 0.0004	R = 0.16, *p* = 0.0042	R = 0.17, *p* = 0.0033		
Surgery duration	R = 0.22, *p* < 0.0001	R = 0.25, *p* < 0.0001	R = 0.26, *p* < 0.0001	R = 0.28, *p* < 0.0001		
Ki-67	R = 0.04, *p* = *0*.4699	R = 0.04, *p* = 0.4381	R = 0.00, *p* = *0*.9538	R = 0.01, *p* = 0.7277		
	**Adverse events at discharge**	**Adverse events at discharge imputed**	**Adverse events at follow-up**	**Adverse events at follow-up imputed**	**Major adverse events**	**Major adverse events imputed**
MCS ≥ 4	OR = 1.90 (1.10 – 3.33, *p* = 0.0143)	OR = 1.89 (1.13 – 3.15, *p* = 0.0157)	OR = 1.83 (1.13 – 3.00, *p* = 0.0093)	OR = 1.72 (1.11 – 2.67, *p* = 0.0198)	OR = 2.08 (0.81 – 6.00, *p* = 0.1006)	OR = 2.13 (0.88 – 5.18, *p* = 0.0947)
Brain invasion	OR = 2.30 (0.33 – 13.97, *p* = 0.2691)	OR = 1.66 (0.73 – 3.79, *p* = 0.2298)	OR = 2.19 (0.36 – 15.28, *p* = 0.2970)	OR = 1.71 (0.94 – 3.12, *p* = 0.1686)	OR = 6.67 (0.59 – 45.16, *p* = 0.0125)	OR = 3.47 (1.10 – 10.97, *p* = 0.0364)

Necrosis	OR = 0.91 (0.34 – 2.19, *p = 0.8217*)	OR = 1.37 (0.76 – 2.48, *p* = 0.2998)	OR = 0.95 (0.42 – 2.08, *p = 0.8945*)	OR = 1.52 (0.94 – 2.45, *p* = 0.1272)	OR = 0.89 (0.10 – 4.09, *p = 0.8846*)	OR = 1.72 (0.59 – 4.98, *p* = 0.3217)
Increased cellularity	OR = 1.48 (0.53 – 3.83, *p = 0.3837*)	OR = 1.14 (0.59– 2.21, *p* = 0.6871)	OR = 1.84 (0.74 – 4.63, *p = 0.1400*)	OR = 1.20 (0.74 – 1.96, *p* = 0.4973)	OR = 1.38 (0.14 – 6.54, *p = 0.6801*)	OR = 1.47 (0.34 – 6.24, *p* = 0.6072)
High n/c ratio	OR = 0.80 (0.19 – 2.62, *p = 0.6937*)	OR = 1.20 (0.63 – 2.27, *p* = 0.5769)	OR = 0.93 (0.30 – 2.68, *p = 0.8907*)	OR = 1.42 (0.86 – 2.33, *p* = 0.2449)	OR = 0.84 (0.02 – 6.08, *p = 0.8724*)	OR = 1.70 (0.59 – 4.86, *p* = 0.3267)
Nucleoli	OR = 1.01 (0.23 – 3.47, *p = 0.9931*)	OR = 1.22 (0.67 – 2.21, *p* = 0.5204)	OR = 1.65 (0.52 – 5.21, *p = 0.3282*)	OR = 1.37 (0.85 – 2.21, *p* = 0.2482)	OR = 1.02 (0.02 – 7.52, *p = 0.9845*)	OR = 2.16 (0.83 – 5.62, *p* = 0.1177)
Patternless growth	OR = 0.79 (0.22 – 2.32, *p = 0.6578*)	OR = 1.07 (0.63 – 1.83, *p* = 0.7956)	OR = 1.19 (0.45 – 3.01, *p = 0.6911*)	OR = 1.19 (0.74 – 1.91, *p* = 0.4932)	OR = 1.46 (0.15 – 6.98, *p = 0.6237*)	OR = 0.80 (0.31 – 2.06, *p* = 0.6443)
Preoperative cortisone	OR = 1.12 (0.63 – 1.98, *p = 0.6692*)	OR = 0.85 (0.43 – 1.68, *p* = 0.6355)	OR = 1.18 (0.70 – 1.99, *p = 0.5066*)	OR = 0.65 (0.36 – 1.19, *p* = 0.1769)	OR = 0.90 (0.29 – 2.45, *p = 0.8320*)	OR = 0.47 (0.11 – 2.03, *p* = 0.3150)
Calcifications	OR = 0.84 (0.38 – 1.75, *p = 0.6336*)	OR = 1.18 (0.73 – 1.90, *p* = 0.5080)	OR = 0.64 (0.31 – 1.25, *p = 0.1612*)	OR = 1.36 (0.89 – 2.09, *p* = 0.1695)	OR = 0.44 (0.05 – 1.85, *p = 0.2562*)	OR = 1.12 (0.51 – 2.45, *p* = 0.7866)
Edema	OR = 1.24 (0.74 – 2.07, *p = 0.3848*)	OR = 1.66 (0.85 – 3.27, *p* = 0.1391)	OR = 1.37 (0.86 – 2.17, *p = 0.1597*)	OR = 1.84 (0.98 – 3.45, *p* = 0.0703)	OR = 1.13 (0.47 – 2.68, *p = 0.7605*)	OR = 0.28 (0.04 – 2.04, *p* = 0.2082)
Bone infiltration	OR = 1.65 (0.77 – 3.39, *p = 0.1454*)	OR = 0.87 (0.27 – 2.80, *p* = 0.8211)	OR = 1.84 (0.91 – 3.76, *p = 0.0654*)	OR = 1.38 (0.54 – 3.56, *p* = 0.5228)	OR = 0.26 (0.01 – 1.65, *p = 0.1561*)	OR = 1.56 (0.34 – 7.16, *p* = 0.5694)
Cysitic tumors	OR = 0.95 (0.22 – 3.23, *p = 0.9248*)	OR = 2.80 (0.38 – 20.63, *p* = 0.3133)	OR = 1.50 (0.48 – 4.71, *p = 0.4263*)	OR = 2.15 (0.34 – 14.83, *p* = 0.5340)	OR = 1.74 (0.18 – 8.27, *p = 0.4733*)	OR = 0.00 (0.00 – inf, *p* = 0.9899)
Parenchymal infiltration	OR = 5.76 (0.30 – 342.44, *p = 0.1069*)	OR = 1.12 (0.51 – 2.44, *p* = 0.7847)	OR = 3.00 (0.15 – 178.09, *p = 0.3472*)	OR = 1.44 (0.73 – 2.83, *p* = 0.3295)	OR = 0.00 (0.00 – 29.25, *p = 0.6128*)	OR = 0.36 (0.05 – 2.67, *p* = 0.3188)
Hyperostosis	OR = 1.07 (0.42 – 2.52, *p = 0.8622*)	OR = 1.23 (0.72 – 2.09, *p* = 0.4447)	OR = 1.45 (0.66 – 3.19, *p = 0.3056*)	OR = 1.44 (0.91 – 2.30, *p* = 0.1561)	OR = 0.35 (0.01 – 2.26, *p = 0.2825*)	OR = 1.23 (0.50 – 3.01, *p* = 0.6534)
Nicotine	OR = 1.25 (0.70 – 2.21, *p = 0.4139*)	OR = 1.89 (1.13 – 3.15, *p* = 0.0157)	OR = 1.47 (0.87 – 2.47, *p = 0.1249*)	OR = 1.72 (1.11 – 2.67, *p* = 0.0198)	OR = 1.21 (0.40 – 3.23, *p* = 0.6891)	OR = 2.13 (0.88 – 5.18, *p* = 0.0947)

Multivariate Imputation by Chained Equations with predictive mean matching for pre- and perioperative risk factors correlated with AE (TDN) at discharge or follow-up (n = 361), and major adverse events. We generated 40 imputed datasets for discharge, follow-up, and major adverse events (seeds: 123 for discharge, 456 for follow-up) with 5 iterations per imputation cycle. Kendall’s Tau (T) and Spearman’s rank correlation coefficient (R) were calculated for each imputed dataset separately and pooled using Fisher's z-transformation: $z = 0.5 \times \ln [(1 + r) / (1 - r)]$. The transformed values were averaged and backtransformed to the reported pooled correlation. Logistic regression was used for binary variables. Odds ratios (OR), confidence intervals, and p-values were pooled across imputations using Rubin’s rule.

Table 4 Pre- and perioperative risk factors correlated with TDN corrected with the Benjamini-Hochberg procedure

Pre- and perioperative factors	TDN at discharge	BH	TDN at follow-up	BH
ASA	T = 0.09, *p* = 0.0673	*p* = 0.0741	T = 0.10, *p* = 0.0335	*p* = 0.0461

MCS	T = 0.15, *p* = 0.0012	*p* = 0.0026	T = 0.13, *p* = 0.0052	*p* = 0.0114
NIHSS	T = 0.11, *p* = 0.0261	*p* = 0.0319	T = 0.10, *p* = 0.0400	*p* = 0.0489
KPS	T = -0.15, *p* = 0.0006	*p* = 0.0017	T = -0.16, *p* = 0.0004	*p* = 0.0021
Age	R = 0.14, *p* = 0.0065	*p* = 0.0089	R = 0.07, *p* = 0.1903	*p* = 0.2094
Tumor diameter	R = 0.20, *p* < 0.0001	*p* = 0.0005	R = 0.17, *p* = 0.0017	*p* = 0.0061
Tumor volume	R = 0.19, *p* = 0.0045	*p* = 0.0070	R = 0.14, *p* = 0.0155	*p* = 0.0249
Simpson grade	T = 0.21, *p* < 0.0001	*p* = 0.0001	T = 0.11, *p* = 0.0159	*p* = 0.0249
Blood loss	R = 0.17, *p* = 0.0024	*p* = 0.0043	R = 0.16, *p* = 0.0042	*p* = 0.0114
Surgery duration	R = 0.22, *p* < 0.0001	*p* = 0.0005	R = 0.26, *p* < 0.0001	*p* = 0.0001
Ki-67	R = 0.04, *p* = 0.4699	*p* = 0.4699	R = 0.00, *p* = 0.9538	*p* = 0.9538

**Cranial pre- and perioperative factors**	**TDN at discharge**	**BH**	**TDN at follow-up**	**BH**
ASA	T = 0.12, *p* = 0.0240	*p* = 0.0264	T = 0.13, *p* = 0.0087	*p* = 0.0191
MCS	T = 0.15, *p* = 0.0012	*p* = 0.0026	T = 0.13, *p* = 0.0052	*p* = 0.0143
NIHSS	T = 0.15, *p* = 0.0027	*p* = 0.0042	T = 0.14, *p* = 0.0047	*p* = 0.0143
KPS	T = -0.19, *p* < 0.0001	*p* = 0.0006	T = -0.19, *p* < 0.0001	*p* = 0.0011
Age	R = 0.17, *p* = 0.0016	*p* = 0.0029	R = 0.11, *p* = 0.0503	*p* = 0.0553
Tumor diameter	R = 0.18, *p* = 0.0010	*p* = 0.0026	R = 0.13, *p* = 0.0177	*p* = 0.0306
Tumor volume	R = 0.16, *p* = 0.0066	*p* = 0.0091	R = 0.13, *p* = 0.0276	*p* = 0.0351
Simpson grade	T = 0.21, *p* < 0.0001	*p* = 0.0006	T = 0.11, *p* = 0.0195	*p* = 0.0306
Blood loss	R = 0.15, *p* = 0.0110	*p* = 0.0134	R = 0.13, *p* = 0.0287	*p* = 0.0351
Surgery duration	R = 0.22, *p* = 0.0003	*p* = 0.0011	R = 0.23, *p* = 0.0002	*p* = 0.0011
Ki-67	R = 0.04, *p* = 0.4436	*p* = 0.4436	R = 0.01, *p* = 0.8942	*p* = 0.8942

Correlations are displayed with Kendall's Tau (T) and Spearman's rank correlation coefficient (R). All reported correlations (n = 11) were included in the Benjamini-Hochberg procedure (BH) for the full cohort at discharge, follow-up (n = 361), cranial tumors (n = 334), and cranial tumors at follow-up (n = 329).

Table 5 Pre- and perioperative risk factors associated with AE corrected with the Benjamini-Hochberg procedure

**Pre- and perioperative factors**	**Adverse events at discharge**	**BH**	**Adverse events at follow-up**	**BH**	**Major adverse events**	**BH**
Multiple tumors	X^2 = 6.64, *p* = 0.0010	*p* = 0.0639	X^2 = 8.03, *p* = 0.0046	*p* = 0.0245	X^2 = 0.00, *p* = 0.9853	*p* = 0.9853

Embolization	$X^2 = 4.86$, $p = 0.0276$	$p = 0.1255$	$X^2 = 5.10$, $p = 0.0239$	$p = 0.0919$	$X^2 = 0.03$, $p = 0.8529$	$p = 0.9761$
Mental alterations	$X^2 = 2.78$, $p = 0.0954$	$p = 0.2347$	$X^2 = 2.64$, $p = 0.1040$	$p = 0.2298$	$X^2 = 11.27$, $p = 0.0008$	$p = 0.0257$
Seizure	$X^2 = 1.16$, $p = 0.2809$	$p = 0.4731$	$X^2 = 4.97$, $p = 0.0258$	$p = 0.0919$	$X^2 = 0.00$, $p = 0.9516$	$p = 0.9853$
Age ≥ 60	$X^2 = 4.63$, $p = 0.0314$	$p = 0.1255$	$X^2 = 1.36$, $p = 0.2436$	$p = 0.3713$	$X^2 = 1.37$, $p = 0.2415$	$p = 0.5857$
MCS ≥ 4	$X^2 = 6.00$, $p = 0.0143$	$p = 0.0763$	$X^2 = 6.77$, $p = 0.0093$	$p = 0.0424$	$X^2 = 2.70$, $p = 0.1006$	$p = 0.3578$
mRS ≥ 2	$X^2 = 8.35$, $p = 0.0039$	$p = 0.0412$	$X^2 = 8.21$, $p = 0.0042$	$p = 0.0245$	$X^2 = 3.84$, $p = 0.0502$	$p = 0.2645$
sex	$X^2 = 3.56$, $p = 0.0594$	$p = 0.1899$	$X^2 = 3.98$, $p = 0.0461$	$p = 0.1414$	$X^2 = 0.05$, $p = 0.8201$	$p = 0.9761$
Recurrent tumors	$X^2 = 7.80$, $p = 0.0052$	$p = 0.0417$	$X^2 = 8.73$, $p = 0.0031$	$p = 0.0245$	$X^2 = 6.75$, $p = 0.0094$	$p = 0.1331$
Radiation induced tumors	$X^2 = 0.65$, $p = 0.4205$	$p = 0.5607$	$X^2 = 3.89$, $p = 0.0486$	$p = 0.1414$	$X^2 = 1.79$, $p = 0.1807$	$p = 0.4819$
WHO CNS grade ≥ 2	$X^2 = 1.13$, $p = 0.2877$	$p = 0.4295$	$X^2 = 2.59$, $p = 0.1077$	$p = 0.2298$	$X^2 = 5.50$, $p = 0.0191$	$p = 0.1524$
Brain invasion	$X^2 = 1.22$, $p = 0.2691$	$p = 0.4494$	$X^2 = 1.09$, $p = 0.2970$	$p = 0.4201$	$X^2 = 6.24$, $p = 0.0125$	$p = 0.1331$
Postoperative cortisone	$X^2 = 12.98$, $p = 0.0003$	$p = 0.0050$	$X^2 = 17.64$, $p < 0.0001$	$p = 0.0009$	$X^2 = 3.66$, $p = 0.0558$	$p = 0.2645$
Simpson grade ≥ 3	$X^2 = 29.62$, $p < 0.0001$	$p < 0.0001$	$X^2 = 10.83$, $p = 0.0010$	$p = 0.0160$	$X^2 = 3.43$, $p = 0.0641$	$p = 0.2645$
Cranial tumors	$X^2 = 3.58$, $p = 0.0585$	$p = 0.1899$	$X^2 = 8.62$, $p = 0.0033$	$p = 0.0245$	$X^2 = 1.09$, $p = 0.2968$	$p = 0.5936$
ASA ≥ 3	$X^2 = 2.05$, $p = 0.1527$	$p = 0.3054$	$X^2 = 3.14$, $p = 0.0766$	$p = 0.1885$	$X^2 = 2.18$, $p = 0.1401$	$p = 0.4483$
Necrosis	$X^2 = 0.05$, $p = 0.8217$	$p = 0.9067$	$X^2 = 0.02$, $p = 0.8945$	$p = 0.8945$	$X^2 = 0.02$, $p = 0.8846$	$p = 0.9761$
Increased cellularity	$X^2 = 0.76$, $p = 0.3837$	$p = 0.5597$	$X^2 = 2.18$, $p = 0.1400$	$p = 0.2489$	$X^2 = 0.17$, $p = 0.6801$	$p = 0.9587$
High n/c ratio	$X^2 = 0.16$, $p = 0.6937$	$p = 0.7928$	$X^2 = 0.02$, $p = 0.8907$	$p = 0.8945$	$X^2 = 0.03$, $p = 0.8724$	$p = 0.9761$
Nucleoli	$X^2 = 0.00$, $p = 0.9931$	$p = 0.9931$	$X^2 = 0.96$, $p = 0.3282$	$p = 0.4201$	$X^2 = 0.00$, $p = 0.9845$	$p = 0.9853$
Patternless growth	$X^2 = 0.20$, $p = 0.6578$	$p = 0.7928$	$X^2 = 0.16$, $p = 0.6912$	$p = 0.7626$	$X^2 = 0.24$, $p = 0.6237$	$p = 0.9504$
Symptomatic tumors	$X^2 = 3.18$, $p = 0.0747$	$p = 0.2083$	$X^2 = 2.26$, $p = 0.1328$	$p = 0.2489$	$X^2 = 0.70$, $p = 0.4015$	$p = 0.7559$
Headache	$X^2 = 3.10$, $p = 0.0781$	$p = 0.2083$	$X^2 = 0.98$, $p = 0.3224$	$p = 0.4201$	$X^2 = 0.53$, $p = 0.4686$	$p = 0.7971$
Focal neurological deficits	$X^2 = 1.48$, $p = 0.2241$	$p = 0.4218$	$X^2 = 0.12$, p = 0.7329	$p = 0.7818$	$X^2 = 3.38$, $p = 0.0661$	$p = 0.2645$
Preoperative cortisone	$X^2 = 0.18$, $p = 0.6692$	$p = 0.7928$	$X^2 = 0.44$, $p = 0.5066$	$p = 0.5790$	$X^2 = 2.42$, $p = 0.8320$	$p = 0.9761$
Calcifications	$X^2 = 0.23$, $p = 0.6336$	$p = 0.7928$	$X^2 = 1.96$, $p = 0.1612$	$p = 0.2580$	$X^2 = 1.29$, $p = 0.2562$	$p = 0.5857$
Edema	$X^2 = 0.76$, $p = 0.3848$	$p = 0.5597$	$X^2 = 1.98$, $p = 0.1597$	$p = 0.2580$	$X^2 = 0.09$, $p = 0.7605$	$p = 0.9761$
Bone infiltration	$X^2 = 2.12$, $p = 0.1454$	$p = 0.3054$	$X^2 = 3.39$, $p = 0.065$	$p = 0.1744$	$X^2 = 2.01$, p = 0.1561	$p = 0.4541$
Cysitic tumors	$X^2 = 0.01$, $p = 0.9248$	$p = 0.9546$	$X^2 = 0.63$, $p = 0.4263$	$p = 0.5052$	$X^2 = 0.51$, $p = 0.4733$	$p = 0.7971$
Parenchymal infiltration	$X^2 = 2.60$, $p = 0.1069$	$p = 0.2444$	$X^2 = 0.88$, $p = 0.3472$	$p = 0.4273$	$X^2 = 0.26$, $p = 0.6128$	$p = 0.9504$
Hyperostosis	$X^2 = 0.03$, $p = 0.8622$	$p = 0.9196$	$X^2 = 1.05$, $p = 0.3056$	$p = 0.4201$	$X^2 = 1.16$, $p = 0.2825$	$p = 0.5936$
Nicotine	$X^2 = 0.67$, p = 0.4139	$p = 0.5607$	$X^2 = 2.35$, p = 0.1249	$p = 0.2489$	$X^2 = 0.16$, p = 0.6891	$p = 0.9587$
**Cranial pre- and perioperative factors**	**Adverse events at discharge**	**BH**	**Adverse events at follow-up**	**BH**	**Major adverse events**	**BH**

Multiple tumors	X^2 = 6.46, *p* = 0.0110	*P = 0.0762*	X^2 = 7.61, *p* = 0.0058	*p* = 0.0507	X^2 = 0.00, *p* = 0.9812	*p = 0.99*
Embolization	X^2 = 4.28, *p* = 0.0386	*p = 0.1503*	X^2 = 4.33, *p* = 0.0374	*p* = 0.1636	X^2 = 0.02, *p* = 0.9008	*p = 0.99*
Mental alterations	X^2 = 1.94, *p* = 0.1635	*p = 0.3816*	X^2 = 1.47, *p* = 0.2247	*p* = 0.4138	X^2 = 9.94, *p* = 0.0016	*p = 0.0351*
Headache	X^2 = 4.36, *p* = 0.0367	*p = 0.1503*	X^2 = 2.27, *p* = 0.1317	*p* = 0.3515	X^2 = 0.80, *p* = 0.3702	*p = 0.6819*
Seizure	X^2 = 0.64, *p* = 0.4235	*p = 0.6375*	X^2 = 3.28, *p* = 0.0702	*p* = 0.2492	X^2 = 0.01, *p* = 0.9294	*p = 0.99*
Age ≥ 60	X^2 = 5.89, *p* = 0.0153	*p = 0.0762*	X^2 = 2.17, *p* = 0.1406	*p* = 0.3515	X^2 = 2.60, *p* = 0.1068	*p = 0.3114*
MCS ≥ 4	X^2 = 6.00, *p* = 0.0143	*p = 0.0762*	X^2 = 6.77, *p* = 0.0093	*p* = 0.0548	X^2 = 2.70, *p* = 0.1006	*p = 0.3114*
ASA ≥ 3	X^2 = 3.50, *p* = 0.0613	*p = 0.2146*	X^2 = 4.98, *p* = 0.0257	*p* = 0.1284	X^2 = 3.24, *p* = 0.0721	*p = 0.2522*
mRS ≥ 2	X^2 = 11.45, *p* = 0.0007	*p = 0.0125*	X^2 = 11.39, *p* = 0.0007	*p* = 0.0129	X^2 = 5.30, *p* = 0.0214	*p = 0.1246*
Sex	X^2 = 1.63, *p* = 0.2017	*p = 0.4412*	X^2 = 1.82, *p* = 0.1770	*p* = 0.3872	X^2 = 0.02, *p* = 0.8802	*p = 0.99*
Recurrent tumors	X^2 = 6.47, *p* = 0.0110	*p = 0.0762*	X^2 = 6.75, *p* = 0.0094	*p* = 0.0548	X^2 = 5.88, *p* = 0.0153	*p = 0.1133*
Brain invasion	X^2 = 1.14, *p* = 0.2850	*p = 0.5542*	X^2 = 0.94, *p* = 0.3311	*p* = 0.5039	X^2 = 6.08, *p* = 0.0136	*p* = 0.1133
WHO CNS grade ≥ 2	X^2 = 0.83, *p* = 0.3636	*p = 0.6362*	X^2 = 1.48, *p* = 0.2238	*p* = 0.4138	X^2 = 5.78, *p* = 0.0162	*p = 0.1133*
Postoperative cortisone	X^2 = 9.36, *p* = 0.0022	*p = 0.0259*	X^2 = 13.84, *p* = 0.0002	*p = 0.0070*	X^2 = 3.99, *p* = 0.0457	*p = 0.2283*
Simpson Grade ≥ 3	X^2 = 27.05, *p* < 0.0001	*p < 0.001*	X^2 = 8.73, *p* = 0.0031	*p* = 0.0364	X^2 = 3.24, *p* = 0.0721	*p = 0.2522*
Infratentorial tumors	X^2 = 0.85, *p* = 0.3574	*p = 0.6362*	X^2 = 0.30, *p* = 0.5865	*p* = 0.6925	X^2 = 1.34, *p* = 0.2474	*p = 0.5239*
Parasagittal tumors	X^2 = 0.02, *p* = 0.8966	*p = 0.9656*	X^2 = 0.28, *p* = 0.5936	*p* = 0.6925	X^2 = 0.03, *p* = 0.8659	*p = 0.5239*
Skull base	X^2 = 0.05, *p* = 0.8315	*p = 0.9388*	X^2 = 0.14, *p* = 0.7093	*p* = 0.7757	X^2 = 0.66, *p* = 0.4182	*p = 0.6971*
Transsphenoidal surgery	X^2 = 2.14, *p* = 0.1434	*p = 0.3585*	X^2 = 1.38, *p* = 0.2404	*p* = 0.4207	X^2 = 9.55, *p* = 0.0020	*p = 0.0351*
Radiation induced	X^2 = 0.48, *p* = 0.4897	*p = 0.6856*	X^2 = 3.26, *p* = 0.0712	*p* = 0.2492	X^2 = 0.52, *p* = 0.4712	*p = 0.5239*
Necrosis	X^2 = 0.05, *p* = 0.8215	*p = 0.9388*	X^2 = 0.04, *p* = 0.8446	*p* = 0.8695	X^2 =0.02, *p* = 0.8825	*p = 0.99*
Increased cellularity	X^2 = 0.31, *p* = 0.5767	*p = 0.7763*	X^2 = 1.56, *p* = 0.2111	*p* = 0.4138	X^2 = 0.25, *p* = 0.6199	*p = 0.8678*
High n/c ratio	X^2 = 0.60, *p* = 0.4372	*p = 0.6375*	X^2 = 0.17, *p* = 0.6816	*p* = 0.7696	X^2 = 0.01, *p* = 0.9416	*p = 0.99*
Nucleoli	X^2 = 0.00, *p* = 0.9478	*p = 0.9757*	X^2 = 0.69, *p* = 0.4066	*p* = 0.5473	X^2 = 0.00, *p* = 0.9900	*p = 0.99*
Patternless growth	X^2 = 0.64, *p* = 0.4224	*p = 0.6375*	X^2 = 0.02, *p* = 0.8895	*p* = 0.8895	X^2 = 0.34, *p* = 0.5608	*p = 0.8534*
Symptomatic tumors	X^2 = 3.26, *p* = 0.0708	*p = 0.2254*	X^2 = 2.45, *p* = 0.1172	*p* = 0.3515	X^2 = 0.74, *p* = 0.3910	*p = 0.6842*
Focal neurological deficits	X^2 = 2.92, *p* = 0.0877	*p = 0.2558*	X^2 = 1.13, *p* = 0.2882	*p* = 0.4584	X^2 = 3.63, *p* = 0.0568	*p = 0.2484*
Preoperative cortisone	X^2 = 0.01, *p* = 0.9104	*p = 0.9656*	X^2 = 0.11, *p* = 0.7422	*p* = 0.7872	X^2 = 0.06, *p* = 0.8072	*p = 0.99*
Calcifications	X^2 = 0.06, *p* = 0.8014	*p = 0.9388*	X^2 = 1.30, *p* = 0.2548	*p* = 0.4246	X^2 = 1.09, *p* = 0.2975	*p = 0.5785*
Edema	X^2 = 0.22, *p* = 0.6403	*p = 0.8301*	X^2 = 0.70, *p* = 0.4021	*p* = 0.5473	X^2 = 0.02, *p* = 0.9018	*p = 0.99*
Bone infiltration	X^2 = 1.52, *p* = 0.2174	*p = 0.4476*	X^2 = 2.36, *p* = 0.1244	*p* = 0.3515	X^2 = 2.22, *p* = 0.1359	*p = 0.3659*
Cysitic tumors	X^2 = 0.05, *p* = 0.8281	*p* = 0.9388	X^2 = 0.37, *p* = 0.5431	*p* = 0.6789	X^2 = 0.42, *p* = 0.5172	*p = 0.8228*
Parenchymal infiltration	X^2 = 2.35, *p* = 0.1249	*p* = 0.3363	X^2 = 0.73, *p* = 0.3919	*p* = 0.5473	X^2 = 0.27, *p* = 0.6036	*p = 0.8678*

Hyperostosis	$X^2 = 0.00$, $p =$ 0.9907	$p =$ 0.9907	$X^2 = 0.57$, $p =$ 0.4514	$p =$ 0.5851	$X^2 = 1.30$, p = 0.2545	$p =$ *0.5239*
Nicotine	$X^2 = 0.72$, $p =$ 0.3963	$p =$ 0.6375	$X^2 = 2.06$, $p =$ 0.1508	$p =$ 0.3519	$X^2 = 0.16$, p = 0.6875	$p =$ *0.9255*

Pre- and perioperative factors association with adverse events at discharge, follow-up (n = 361), and major adverse events are reported with Chi-Square (X^2). The Benjamini–Hochberg (BH) procedure was applied to the full cohort at discharge and follow-up (n = 361), to patients with cranial tumors (n = 334), and to the cranial tumor subgroup at follow-up (n = 329). A total of 31 factors were included in the BH analysis for the full cohort and 35 for the cranial tumor subgroup. ASA = American Society of Anesthesiologists risk classification, MCS = Milan Complexity Score, NIHSS = National Institute of Health Stroke Scale, KPS = Karnofsky Performance Scale, mRS = modified Rankin Scale, n/c ratio = nucleus-to-cytoplasmic ratio.

Table 6 Pre- and perioperative risk factors insignificantly associated with AE (TDN)

**Pre- and perioperative factors**	**TDN**	**TDN at follow-up**	
Ki-67	R = 0.04, *p = 0*.4699	R = 0.00, *p = 0*.9538	
	**Adverse events at discharge**	**Adverse events at follow-up**	**Major adverse events**
ASA ≥ 3	OR = 1.41 (0.85 – 2.34, *p = 0.1527*)	OR = 1.48 (0.94 – 2.34, *p = 0.0766*)	OR = 1.78 (0.76 – 4.17, *p = 0.1401*)
Necrosis	OR = 0.91 (0.34 – 2.19, *p = 0.8217*)	OR = 0.95 (0.42 – 2.08, *p = 0.8945*)	OR = 0.89 (0.10 – 4.09, *p = 0.8846*)
Increased cellularity	OR = 1.48 (0.53 – 3.83, *p = 0.3837*)	OR = 1.84 (0.74 – 4.63, *p = 0.1400*)	OR = 1.38 (0.14 – 6.54, *p = 0.6801*)
High n/c ratio	OR = 0.80 (0.19 – 2.62, *p = 0.6937*)	OR = 0.93 (0.30 – 2.68, *p = 0.8907*)	OR = 0.84 (0.02 – 6.08, *p = 0.8724*)
Nucleoli	OR = 1.01 (0.23 – 3.47, *p = 0.9931*)	OR = 1.65 (0.52 – 5.21, *p = 0.3282*)	OR = 1.02 (0.02 – 7.52, *p = 0.9845*)
Patternless growth	OR = 0.79 (0.22 – 2.32, *p = 0.6578*)	OR = 1.19 (0.45 – 3.01, *p = 0.6911*)	OR = 1.46 (0.15 – 6.98, *p = 0.6237*)
Symptomatic tumors	OR = 1.91 (0.91 – 4.42, *p = 0.0747*)	OR = 1.58 (0.84 – 3.07, *p = 0.1328*)	OR = 1.68 (0.49 – 9.00, *p = 0.4015*)
Headache	OR = 0.59 (0.30 – 1.09, *p = 0.0781*)	OR = 0.78 (0.45 – 1.32, *p = 0.3224*)	OR = 0.69 (0.20 – 1.95, *p = 0.4686*)
Focal neurological deficits	OR = 1.34 (0.81 – 2.19, *p = 0.2241*)	OR = 1.08 (0.69 – 1.68, *p = 0.7329*)	OR = 2.06 (0.88 – 5.03, *p = 0.0661*)
Preoperative cortisone	OR = 1.12 (0.63 – 1.98, *p = 0.6692*)	OR = 1.18 (0.70 – 1.99, *p = 0.5066*)	OR = 0.90 (0.29 – 2.45, *p = 0.8320*)
Calcifications	OR = 0.84 (0.38 – 1.75, *p = 0.6336*)	OR = 0.64 (0.31 – 1.25, *p = 0.1612*)	OR = 0.44 (0.05 – 1.85, *p = 0.2562*)
Edema	OR = 1.24 (0.74 – 2.07, *p = 0.3848*)	OR = 1.37 (0.86 – 2.17, *p = 0.1597*)	OR = 1.13 (0.47 – 2.68, *p = 0.7605*)
Bone infiltration	OR = 1.65 (0.77 – 3.39, *p = 0.1454*)	OR = 1.84 (0.91 – 3.76, *p = 0.0654*)	OR = 0.26 (0.01 – 1.65, *p = 0.1561*)
Cysitic tumors	OR = 0.95 (0.22 – 3.23, *p = 0.9248*)	OR = 1.50 (0.48 – 4.71, *p = 0.4263*)	OR = 1.74 (0.18 – 8.27, *p = 0.4733*)
Parenchymal infiltration	OR = 5.76 (0.30 – 342.44, *p = 0.1069*)	OR = 3.00 (0.15 – 178.09, *p = 0.3472*)	OR = 0.00 (0.00 – 29.25, *p = 0.6128*)
Hyperostosis	OR = 1.07 (0.42 – 2.52, *p = 0.8622*)	OR = 1.45 (0.66 – 3.19, *p = 0.3056*)	OR = 0.35 (0.01 – 2.26, *p = 0.2825*)
Nicotine	OR = 1.25 (0.70 – 2.21, *p = 0.4139*)	OR = 1.47 (0.87 – 2.47, *p = 0.1249*)	OR = 1.21 (0.40 – 3.23, *p* = 0.6891)

Pre- and perioperative factors with no significant association with adverse events and therapy-disability-neurology grading (TDN) at discharge or follow-up (n = 361), and major adverse events. Correlations are

displayed with Kendall's Tau (T); Spearman's rank correlation coefficient (R) associations are reported as odds ratios (OR) with confidence intervals. ASA = American Society of Anesthesiologists risk classification, n/c ratio = nucleus-to-cytoplasmic ratio.

Table 7 Pre- and perioperative risk factors associated with AE reported with odds ratios for cranial tumors

	**Adverse events at discharge**	**Adverse events at follow-up**	**Major adverse events**
Multiple tumors	OR = 2.21 (1.12 – 4.30, *p* = 0.0110)	OR = 2.33 (1.21 – 4.57, *p* = 0.0058)	OR = 0.99 (0.24 – 3.08, *p* = 0.9812)
Embolization	OR = 3.35 (0.83 – 14.23, *p* = 0.0386)	OR = 3.79 (0.89 – 22.60, *p* = 0.0374)	OR = 1.14 (0.03 – 8.61, *p* = 0.9008)
Mental alterations	OR = 1.52 (0.79 – 2.85, *p* = 0.1635)	OR = 1.41 (0.78 – 2.56, *p* = 0.2247)	OR = 3.52 (1.39 – 8.63, *p* = 0.0016)
Headache	OR = 0.53 (0.27 – 0.99, *p* = 0.0367)	OR = 0.68 (0.39 – 1.16, *p* = 0.1317)	OR = 0.63 (0.18 – 1.80, *p* = 0.3702)
Seizure	OR = 1.27 (0.66 – 2.38, *p* = 0.4235)	OR = 1.66 (0.92 – 3.00, *p* = 0.0702)	OR = 0.96 (0.27 – 2.73, *p* = 0.9294)
Age ≥ 60	OR = 1.83 (1.09 – 3.11, *p* = 0.0152)	OR = 1.39 (0.88 – 2.21, *p* = 0.1406)	OR = 1.96 (0.81 – 5.11, *p* = 0.1068)
MCS ≥ 4	OR = 1.90 (1.10 – 3.33, *p* = 0.0143)	OR = 1.83 (1.13 – 3.00, *p* = 0.0093)	OR = 2.08 (0.81 – 6.00, *p* = 0.1006)
ASA ≥ 3	OR = 1.60 (0.94 – 2.69, *p* = 0.0613)	OR = 1.68 (1.04 – 2.72, *p* = 0.0257)	OR = 2.04 (0.85 – 4.90, *p* = 0.0721)
mRS ≥ 2	OR = 2.34 (1.38 – 3.99, *p* = 0.0007)	OR = 2.24 (1.36 – 3.70, *p* = 0.0007)	OR = 2.47 (1.03 – 5.96, *p* = 0.0214)
Male sex	OR = 1.39 (0.81 – 2.37, *p* = 0.2017)	OR = 1.38 (0.84 – 2.26, *p* = 0.1770)	OR = 1.07 (0.41 – 2.61, *p* = 0.8802)
Recurrent tumors	OR = 2.25 (1.12 – 4.45, *p* = 0.0110)	OR = 2.26 (1.16 – 4.52, *p* = 0.0094)	OR = 2.88 (1.02 – 7.48, *p* = 0.0153)
Brain invasion	OR = 2.24 (0.32 – 13.65, *p* = 0.2850)	OR = 2.08 (0.34 – 14.55, *p* = 0.3311)	OR = 6.58 (0.58 – 44.87, *p* = 0.0136)
WHO CNS grade ≥ 2	OR = 1.39 (0.62 – 2.97, *p* = 0.3636)	OR = 1.51 (0.73 – 3.13, *p* = 0.2238)	OR = 2.99 (0.99 – 8.12, *p* = 0.0162)
Postoperative cortisone	OR = 2.21 (1.29 – 3.86, *p* = 0.0022)	OR = 2.37 (1.46 – 3.88, *p* = 0.0002)	OR = 2.59 (0.93 – 8.31, *p* = 0.0457)
Simpson Grade ≥ 3	OR = 3.65 (2.15 – 6.25, *p* = 0.0000)	OR = 1.98 (1.22 – 3.21, *p* = 0.0031)	OR = 2.04 (0.85 – 4.90, *p* = 0.0721)
Infratentorial tumors	OR = 1.36 (0.66 – 2.71, *p* = 0.3574)	OR = 1.19 (0.61 – 2.29, *p* = 0.5865)	OR = 1.75 (0.55 – 4.82, *p* = 0.2474)
Parasagittal tumors	OR = 1.05 (0.49 – 2.12, *p* = 0.8966)	OR = 0.84 (0.43 – 1.64, *p* = 0.5936)	OR = 0.43 (0.05 – 1.83, *p* = 0.2506)
Skull base	OR = 1.05 (0.63 – 1.76, *p* = 0.8315)	OR = 0.92 (0.58 – 1.46, *p* = 0.7092)	OR = 1.39 (0.58 – 3.42, *p* = 0.4182)
Transsphenoidal surgery	OR = 2.74 (0.50 – 15.03, *p* = 0.1434)	OR = 2.32 (0.44 – 15.19, *p* = 0.2404)	OR = 7.46 (1.09 – 41.02, *p* = 0.0020)
Nicotine	OR = 1.27 (0.70 – 2.27, *p* = 0.3963)	OR = 1.45 (0.84 – 2.47, *p* = 0.1508)	OR = 1.21 (0.40 – 3.30, *p* = 0.6875)
Radiation induced	OR = 1.55 (0.32 – 6.27, *p* = 0.4897)	OR = 3.29 (0.74 – 20.10, *p* = 0.0712)	OR = 2.64 (0.26 – 13.78, *p* = 0.2115)
Necrosis	OR = 0.91 (0.34 – 2.21, *p* = 0.8215)	OR = 0.93 (0.41 – 2.06, *p* = 0.8446)	OR = 0.89 (0.09 – 4.13, *p* = 0.8825)
Increased cellularity	OR = 1.30 (0.43 – 3.56, *p* = 0.5767)	OR = 1.72 (0.66 – 4.51, *p* = 0.2111)	OR = 1.47 (0.15 – 7.14, *p* = 0.6199)
High n/c ratio	OR = 0.61 (0.11 – 2.27, *p* = 0.4372)	OR = 0.81 (0.24 – 2.48, *p* = 0.6816)	OR = 0.93 (0.02 – 6.82, *p* = 0.9416)
Nucleoli	OR = 0.96 (0.22 – 3.33, *p* = 0.9478)	OR = 1.53 (0.48 – 4.86, *p* = 0.4066)	OR = 0.99 (0.02 – 7.33, *p* = 0.9900)
Patternless growth	OR = 0.63 (0.15 – 2.03, *p* = 0.4224)	OR = 1.06 (0.38 – 2.82, *p* = 0.8895)	OR = 1.58 (0.16 – 7.67, *p* = 0.5608)

Symptomatic tumors	OR = 1.94 (0.91 – 4.52, *p* = 0.0708)	OR = 1.62 (0.85 – 3.19, *p* = 0.1172)	OR = 1.71 (0.49 – 9.16, *p* = 0.3910)
Focal neurological deficits	OR = 1.52 (0.91 – 2.55, *p* = 0.0877)	OR = 1.27 (0.80 – 2.03, *p* = 0.2882)	OR = 2.14 (0.90 – 5.29, *p* = 0.0568)
Preoperative cortisone	OR = 1.03 (0.57 – 1.85, *p* = 0.9104)	OR = 1.09 (0.63 – 1.86, *p* = 0.7422)	OR = 0.89 (0.28 – 2.44, *p* = 0.8072)
Calcifications	OR = 0.91 (0.41 – 1.93, *p* = 0.8014)	OR = 0.69 (0.33 – 1.37, *p* = 0.2548)	OR = 0.46 (0.05 – 1.99, *p* = 0.2975)
Edema	OR = 1.12 (0.66 – 1.90, *p* = 0.6403)	OR = 1.21 (0.75 – 1.95, *p* = 0.4021)	OR = 1.05 (0.43 – 2.54, *p* = 0.9018)
Bone infiltration	OR = 1.53 (0.72 – 3.16, *p* = 0.2174)	OR = 1.67 (0.82 – 3.42, *p* = 0.1244)	OR = 0.24 (0.01 – 1.56, *p* = 0.1359)
Cysitic tumors	OR = 0.88 (0.20 – 3.01, *p* = 0.8281)	OR = 1.36 (0.43 – 4.29, *p* = 0.5431)	OR = 1.65 (0.17 – 7.88, *p* = 0.5172)
Parenchymal infiltration	OR = 5.38 (0.28 – 319.96, *p* = 0.1249)	OR = 2.74 (0.14 – 162.85, *p* = 0.3919)	OR = 0.00 (0.00 – 27.82, *p* = 0.6036)
Hyperostosis	OR = 1.00 (0.39 – 2.34, *p* = 0.9907)	OR = 1.32 (0.60 – 2.90, *p* = 0.4514)	OR = 0.33 (0.01 – 2.13, *p* = 0.2545)

Pre- and perioperative factors were tested for association with adverse events at discharge or follow-up, and major adverse events filtered for cranial tumors (n = 334) and cranial tumors at follow-up (n = 329). Associations are reported as odds ratios (OR) with confidence intervals. ASA = American Society of Anesthesiologists risk classification, MCS = Milan complexity Score, NIHSS = National Institute of Health Stroke Scale, KPS = Karnofsky Performance Scale, mRS = modified Rankin Scale.

Table 8 Length of stay for therapy-disability-neurology grade 0 - 5

**TDN**	**LOS (Mean)**	**Z value (p)**	**LOS (SD)**	**LOS (Median)**	**LOS (IQR)**
0	7.67		3.87	7.0	3.0
1	8.42	- 1.74, *p* = *0.*6189	2.15	8.0	1.25
2	10.5	- 5.18, *p* < 0.0001	4.44	10.0	6.0
3	19.3	- 5.56, *p* < 0.0001	21.1	14.0	7.0
4	42.0	- 4.68, *p* < 0.0001	15.8	44.5	18.2
5	12.5	- 1.53, *p* = *0.*9404	8.74	11.5	7.5

Length of hospital stay reported for TDN 0 - 5. TDN = therapy-disability-neurology grading, Z values, and p values ranked against TDN 0 with Bonferroni correction.

Table 9 Discharge modalities for therapy-disability-neurology grade 0 - 4

**TDN**	**Home**	**Rehabilitation facility**
0	n = 173 (63.60%)	n = 96 (35.29%)
1	n = 3 (23.08%)	n = 10 (76.92%)
2	n = 18 (33.33%)	n = 36 (66.67%)
3	n = 5 (27.78%)	n = 13 (72.22%)
4	n = 0 (0.00%)	n = 6 (100.00%)

Discharge modality reported for TDN 0 - 4. TDN = therapy-disability-neurology grading.

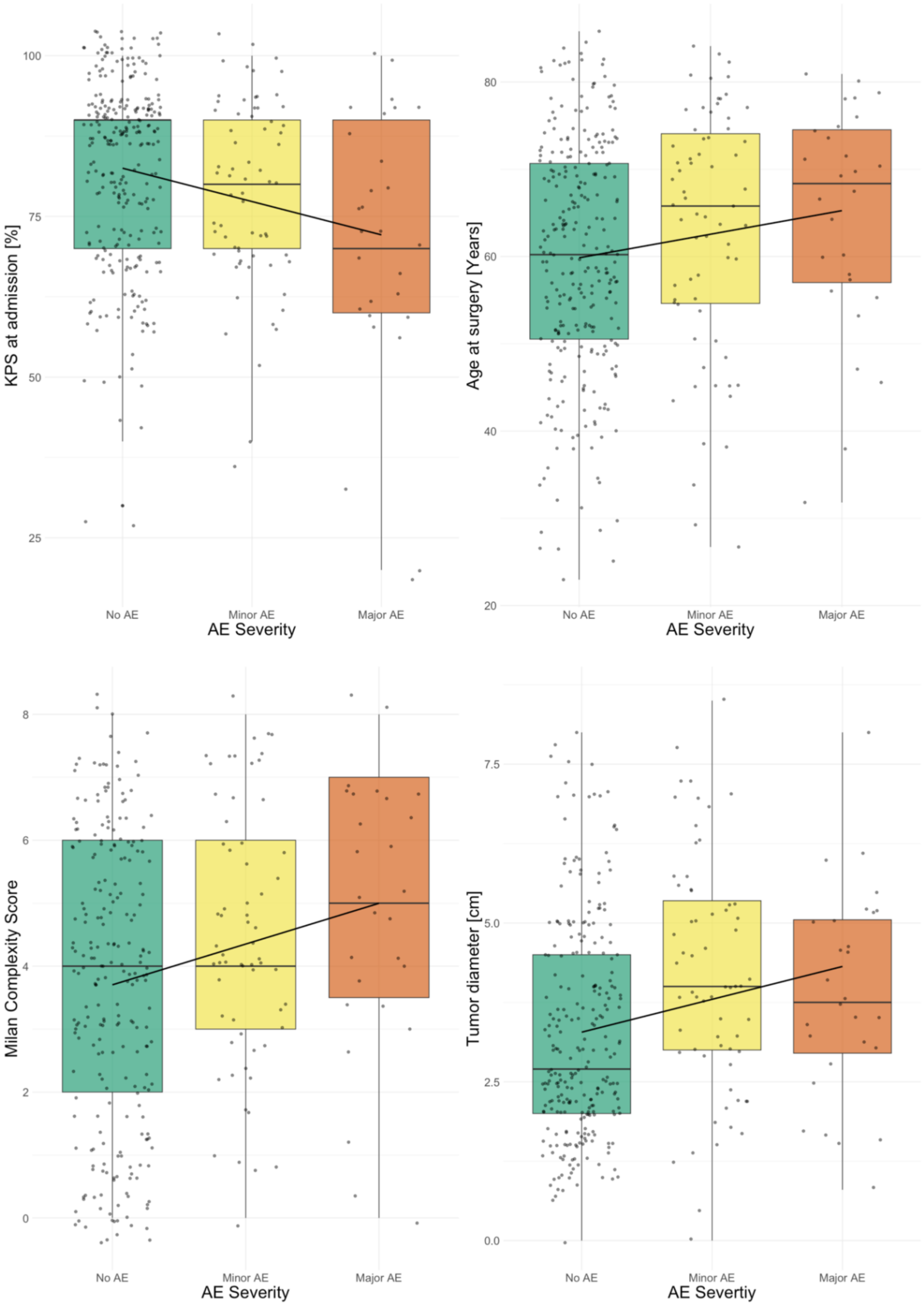
100
75
50
25
KPS at admission [%]
No AE
Minor AE
Major AE
AE Severity
80
60
40
20
Age at surgery [Years]
No AE
Minor AE
Major AE
AE Severity
8
6
4
2
0
Milan Complexity Score
No AE
Minor AE
Major AE
AE Severity
7.5
5.0
2.5
0.0
Tumor diameter [cm]
No AE
Minor AE
Major AE
AE Severtiy

Fig. 1 Relationship between adverse event (AE) severity and preoperative variables

The central line within each box represents the median for each AE severity, while the boxes show the interquartile range (IQR), whiskers extend to 1.5 times the IQR. A linear regression line (solid black) is included. Individual data is visualized as dots and jittered for better interpretability. Max. diameter was capped at 8.6 cm, excluding 6 measurements. KPS = Karnofsky Performance Scale.

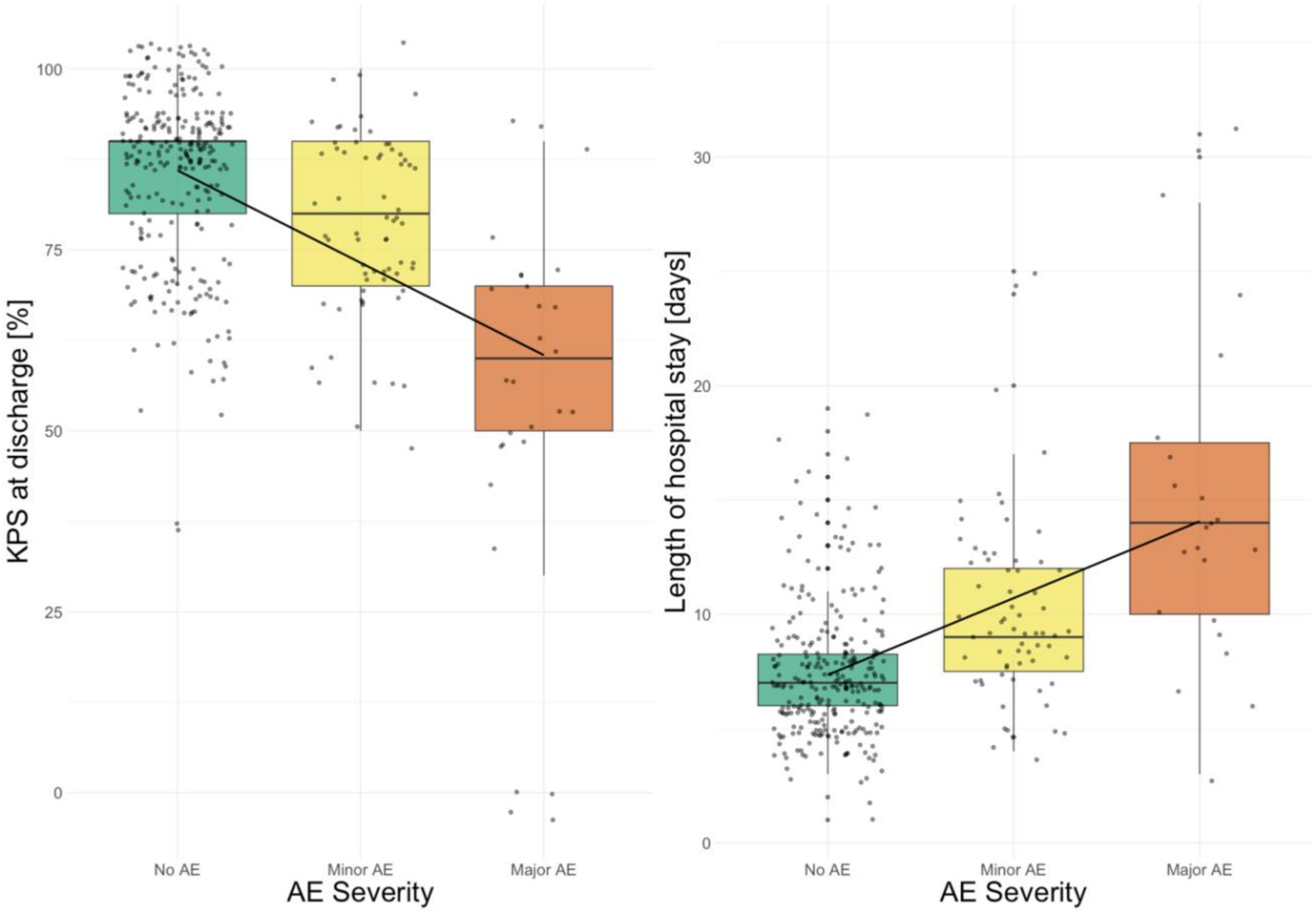

Fig. 2 Relationship between adverse event (AE) severity and outcomes

The central line within each box represents the median for each AE severity, while the boxes show the interquartile range (IQR), whiskers extend to 1.5 times the IQR. A linear regression line (solid black) is included. The individual data is visualized as dots and jittered. Length of hospital stay is capped at 35 for better interpretability, excluding 5 measurements. KPS = Karnofsky Performance Scale.

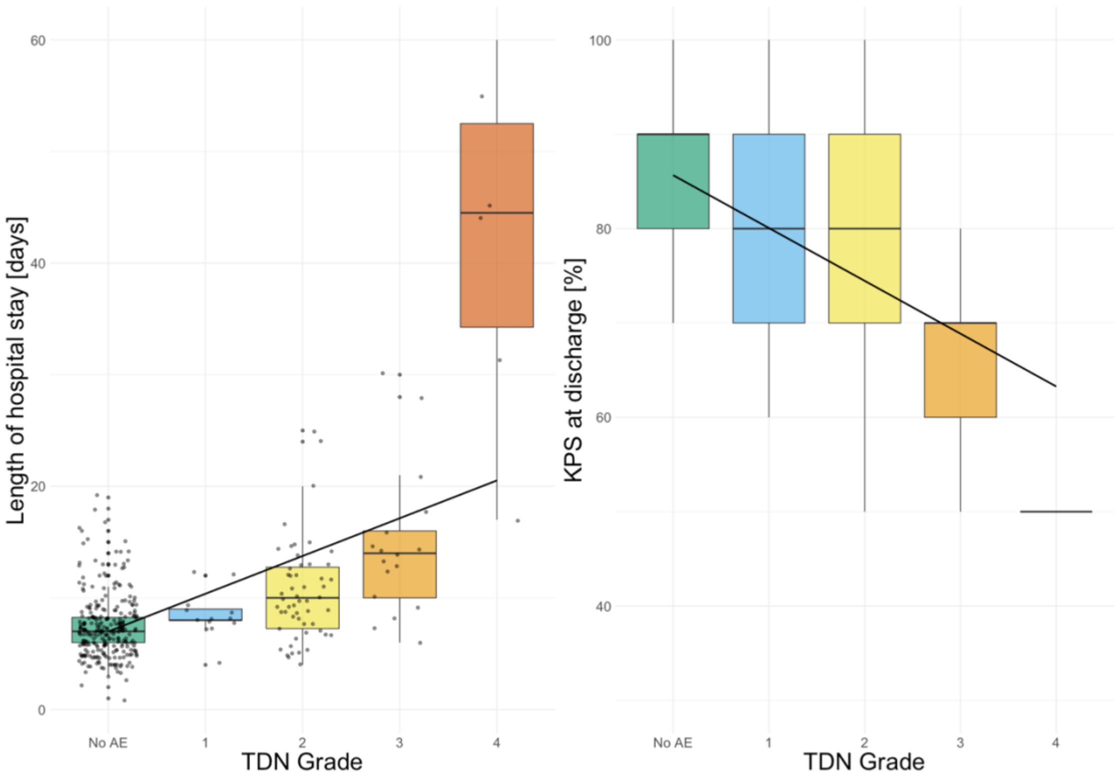

Fig. 3 Relationship between therapy-disability-neurology grading (TDN) and outcomes

TDN 5 (death) was excluded. The central line within each box represents the median LOS/KPS for each TDN, while the boxes show the interquartile range (IQR), whiskers extend to 1.5 times the IQR. A linear regression line (solid black) is included. For LOS, the individual data is visualized as dots and jittered, and one measurement of TDN 3, LOS = 100, is excluded for better interpretability. KPS = Karnofsky Performance Scale.
